# Effect of Various Types of Remineralizing Agents on the Remineralization of Mild and Moderate Defects Caused by Molar-Incisor Hypomineralization in Mexican Schoolchildren: A 12-month Follow-up Randomized Clinical Trial

**DOI:** 10.3290/j.ohpd.c_2796

**Published:** 2026-09-08

**Authors:** Alvaro García Pérez, Danely Minelli Muñiz Alquisiras, Jacqueline Adelina Rodríguez Chávez, Hugo Marcelo Flores Ruíz, Karen Angelina Mora Navarrete

**Affiliations:** a Alvaro García Pérez Professor, Laboratory of Public Health Research, Faculty of Higher Studies (FES), Iztacala, National Autonomous University of Mexico (UNAM), Tlalnepantla, State of Mexico, Mexico. Designed the study, contributed to data analysis, led the writing, reviewed the manuscript, contributed to the discussion.; b Danely Minelli Muñiz Alquisiras Graduate Student, Laboratory of Public Health Research, Faculty of Higher Studies (FES), Iztacala, National Autonomous University of Mexico (UNAM), Tlalnepantla, State of Mexico, Mexico. Contributed to data analysis, reviewed the manuscript, and contributed to the discussion.; c Jacqueline Adelina Rodríguez Chávez Professor, Department of Comprehensive Dental Clinics, University Center for Health Sciences, University of Guadalajara, Guadalajara, Jalisco, México. Reviewed the manuscript, contributed to the discussion.; d Hugo Marcelo Flores Ruíz Professor, Department of Natural and Exact Sciences, University of Guadalajara (CUValles Campus), Guadalajara, Jalisco, México. Reviewed the manuscript, contributed to the discussion.; e Karen Angelina Mora Navarrete Professor, Laboratory of Public Health Research, Faculty of Higher Studies (FES), Iztacala, National Autonomous University of Mexico (UNAM), Tlalnepantla, State of Mexico, Mexico. Reviewed the manuscript, contributed to the discussion.

**Keywords:** children, fluoride varnish, laser fluorescence, Molar-Incisor Hypomineralization, remineralization.

## Abstract

**Purpose:**

Using Laser-Induced Fluorescence (LIF), this study aimed to evaluate the effect of three remineralizing agents on permanent molars and incisors with both mild and moderate Molar-Incisor Hypomineralization (MIH) in 8-to-12-year-old Mexican schoolchildren.

**Materials and Methods:**

In this randomized clinical trial, 104 children were selected to participate and then divided randomly into four groups: Group I: control, Oral-B kids (toothpaste); Group II: Fluor Protector (varnish); Group III: Clinpro White Varnish; and Group IV: MI Paste (CCP-ACP). Both varnishes were applied once every four weeks for twelve months for each group. Remineralization was evaluated by LIF at intervals of 1, 3, 6, 9, and 12 months. Paired-sample t-tests and repeated-measures ANOVA were performed, while the Greenhouse-Geisser correction was used to evaluate sphericity.

**Results:**

The three test agents used increased the level of remineralization observed at the site of mild and moderate MIH defects over the course of twelve months (p<0.001). A similar decrease in average LIF values was observed across the three treatment groups.

**Conclusions:**

The use of both fluoride varnishes and the CPP-ACP toothpaste was observed to promote the remineralization of both mild and moderate MIH defects.

Recent years have seen the increasing use of minimal-intervention dentistry, which focuses on the least invasive treatment options possible in order to decrease dental tissue loss, by means of a biological or therapeutic approach instead of traditional surgical procedures.^27^

An essential element of a biological approach is the use of remineralizing agents, which can be classified as fluoridated agents produced using a calcium-phosphate or herbal base or a variety of combinations.^8^ Remineralizing agents may prevent damage to and conserve the majority of the tooth structure, not only in teeth with caries^1^ but also in those with Molar-Incisor Hypomineralization (MIH).^14^

Remineralizing agents are substances that help to restore lost essential minerals (e.g., calcium and phosphate) on the tooth surface in order to both strengthen it and prevent caries.^27^ Remineralizing agents control the dynamic balance between demineralization and remineralization, which depends on the oral environment, namely saliva, dental plaque, and the tooth surface. Furthermore, topical and systemic fluorides are used to promote the remineralization of enamel.^26^ For decades, topical fluorides have been used to improve the remineralization process, as they increase the availability of fluoride ions in the saliva and improve the formation of fluorapatite, which is known to present great resistance to acid attack and inhibit bacterial metabolic activity.^2^

Fluoride varnishes have been used as a strategy for decreasing and preventing the incidence of caries. On application, these varnishes adhere to the tooth surface over a prolonged time period, achieving the effective release of fluoride, which facilitates the formation of fluoridated hydroxyapatite and reduces the solubility of enamel in acid.^11^ The use of varnishes such as Clinpro White Varnish, Duraphat, and Fluor Protector has been shown to promote the remineralization of carious lesions;^6,21^ recently, the use of fluoride varnishes has also been recommended for the treatment of MIH by protecting damaged enamel and increasing the mineral content of hypomineralized surfaces. However, evidence regarding the efficacy of these varnishes on teeth with MIH remains limited.^29^

Although fluoride continues to be the best agent for intervening in the remineralization process, other calcium-phosphate–based agents are available in other formulations, such as casein phosphopeptide-amorphous calcium phosphate (CPP-ACP), tri-calcium phosphate (a component of Clinpro, 3M Oral Care; St Paul, MN, USA),^22^ and the self-assembling peptide P11-4 (contained in Curodont Repair Fluoride Plus; Zürich, Switzerland).^10,15^

Although the literature contains few studies evaluating the effect of remineralizing agents on teeth affected by MIH, Olgen et al^23^ found that fluoride varnish and toothpastes containing CPP-ACP increase the remineralization of both creamy-white and yellow-brown defects. Moreover, Biondi et al,^3^ using Duraphat (Colgate; New York, NY,USA), Clinpro, and CPP-ACP, reported the increased remineralization of mild and moderate MIH lesions. As part of efforts to improve the mechanical properties of teeth with MIH, fluoride varnishes and toothpastes containing CPP-ACP can be used to participate in the remineralization process, given that both increase the mineral content of the affected enamel, thus preventing the fractures that present after the tooth eruption process.

The present study used laser-induced fluorescence (LIF) to evaluate the effect of three remineralizing agents, Clinpro White Varnish, Fluor Protector, and MI Paste, on the permanent molars and incisors of 8-to-12-year-old Mexican schoolchildren with mild and moderate MIH. The hypothesis of the study was that the three remineralizing agents of interest are equally effective in remineralizing enamel presenting mild and moderate MIH lesions over a 12-month treatment period.

## MATERIALS AND METHODS

### Trial Design and Settings

This was a four parallel-armed randomized clinical trial that was set up and reported according to the Consolidated Standards of Reporting Trials (CONSORT) statement, from January 2024 to January 2025.

The clinical trial was approved by the Ethics Committee at the Iztacala Faculty of Higher Studies of the National Autonomous University of Mexico (CE/FESI/062022/1520). The trial was preregistered and approved at ClinicalTrials.gov (NCT06362681). Parents/guardians signed informed consent, as did the children, to participate in the study.

### Sample Power Calculation

A sample size of at least 75 teeth (25 for each of the three study groups) was required for the clinical trial, with a power of 0.80, effect size of 1 and significance level (α) of 0.05.^23^ The effect size was determined by the difference between means. The effect size was verified by showing the distance between the means between groups, adjusted for their variance. Therefore, the clinical trial comprised 30 teeth per treatment group during the monitoring period.

### Study Population

One-hundred thiry (130) children enrolled in a public primary school in a district (in which no private schools are located) in the municipality of Ayala, in the state of Morelos, Mexico, were invited to participate in the present study. The following inclusion criteria were applied for the schoolchildren participating in the present study: 8–12 years old; of either gender; at least one erupted permanent molar or incisor with the presence of MIH, with creamy-white and/or yellow-brown lesions and either with or without post-eruptive breakdown of the enamel; no caries; and no previous restorations, according to the European Academy of Paediatric Dentistry (EAPD) criteria.^16^ Only mild and moderate MIH lesions were considered by the present study. The following exclusion criteria were applied: the use of a fixed orthodontic appliance; the presence of a systemic disease; the presence of another developmental defect of enamel such as fluorosis or amelogenesis imperfecta; a history of dental trauma; the presence of an allergy; and uncooperative behavior during the application of the remineralizing agents. Twenty-six (26) children were excluded because they did not meet the inclusion criteria and the total sample comprised 104 children. The response rate was 80%.

### Allocation and Intervention Procedures for the Treatment Groups 

**Fig 1:**
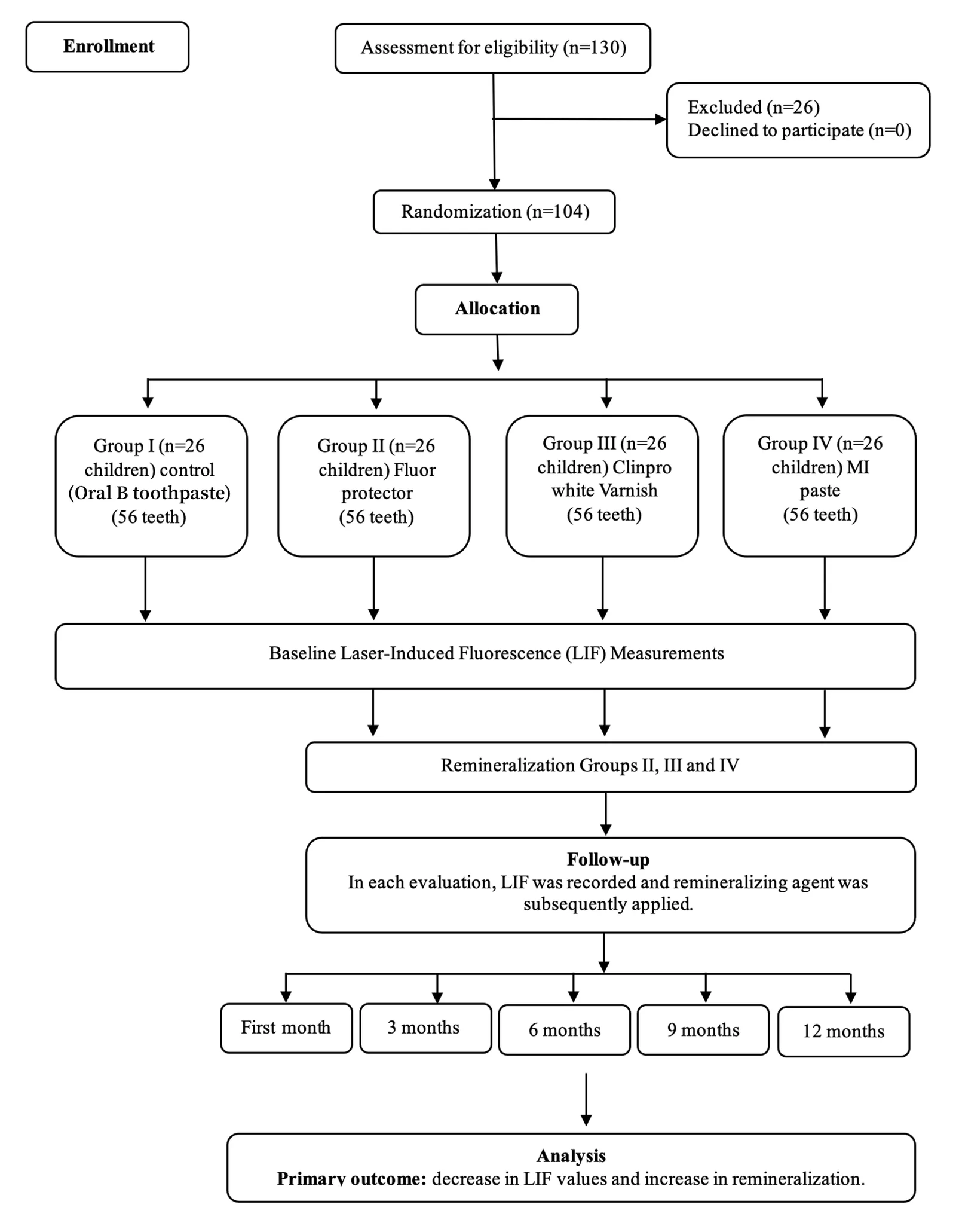
Flowchart for treatment assignment in children with MIH.

In the present study, a block randomization technique was applied to assign each participant to a particular group at a 1:1 allocation ratio, using the online tool found at https://www.randomizer.org. The random allocation sequence was performed by the blinded investigator. The data were captured and coded according to the child’s identification number, while the allocation to each treatment group was blinded. Those children diagnosed with MIH that presented a creamy-white or a yellow-brown color were placed in random order and divided into three treatment groups, while the control group comprised children with teeth presenting healthy enamel. The four groups were as follows: Group I (control), 26 children with 56 healthy teeth: Oral-B Kids toothpaste, sodium fluoride content 1100 ppm (Procter & Gamble; Cincinnati, OH, USA); Group II, 26 children and 56 affected teeth: Fluor Protector, 0.9% difluorosilane on a polyurethane varnish base with ethyl acetate and isoamyl propionate solvents (Ivoclar Vivadent; Schaan, Liechtenstein); Group III, 26 children and 56 affected teeth: Clinpro White Varnish, 5% sodium fluoride and tricalcium phosphate (3M Oral Care; St Paul, MN, USA); Group IV, 26 children and 56 affected teeth: MI Paste, containing CPP-ACP (GC; Tokyo, Japan)^13^ (Fig 1).

In Groups II and III, both varnishes were applied once every four weeks for twelve months for each group. Fluoride varnishes were only applied to permanent molars and incisors affected by MIH. The teeth and molars presenting MIH were not cleaned professionally prior to the application of the varnishes and, instead, were simply brushed without toothpaste and then dried. For the application of fluoride varnishes (Fluor Protector and Clinpro White Varnish) the manufacturer’s instructions for each varnish were followed.

For group IV, MI Paste, parents were advised to brush their children’s teeth 3 times a day with Oral-B Kids fluoride toothpaste (1110 ppm), after which they applied the MI Paste toothpaste onto the MIH teeth using a cotton swab. As instructed by the manufacturer, patients were asked to not eat, drink, or rinse their mouth for at least half an hour after the application of the toothpaste. MI Paste toothpaste was used in the morning and evening for twelve months from the beginning of the study.^13^ MI Paste was only applied to teeth with MIH defects. To ensure treatment adherence, parents monitored and dosed the MI Paste, and the empty container was then returned to the principal investigator for a full one.

During the study, all the children attended a session on toothbrushing, and all participants in the four treatment groups used fluoride toothpaste (Oral-B Kids; sodium fluoride content 1110 ppm) to maintain their oral hygiene three times per day.^13^

#### Blinding

This study was double-blinded, as the participants and the statistician were blinded to the group divisions. Since the method of application of Fluor Protector differs from that of Clinpro White Varnish and MI Paste, and the form of the commercial products differ, blinding of the primary investigator was not possible.

#### Clinical oral examination

The oral examination was performed by two experienced dentists at the selected school; a mouth mirror and a WHO probe were used. Prior to the examination (which adhered to the corresponding infection control standards) of the children’s oral cavity, the children brushed their teeth to remove plaque or food remnants. The two dentists were previously trained and calibrated for MIH via a process consisting of two steps (theoretical and clinical). Their inter and intra-examiner agreement for MIH corresponded to a Cohen’s kappa coefficient of 0.87.

#### LIF Evaluation

The LIF evaluation was conducted using the DIAGNOdent Pen (2190, KaVo; Biberach, Germany). The DIAGNOdent scores offer the possibility to monitor lesion behavior (0–12 sound, 13–24 enamel and 24–99 dentin involvement). The type A probe was used on occlusal surfaces, while the type B probe was used for smooth tooth surfaces. Therefore, low LIF values reflect dental remineralization. In in-vitro studies, the DIAGNOdent Pen showed a higher level of agreement between measurements compared with the histological standard. Furthermore, a stronger association was demonstrated between the DIAGNOdent pen scores and ICDAS-II scores. Therefore, the use of DIAGNOdent Pen is more sensitive and allows remineralization in tooth enamel to be detected.^19^ All the measurements were taken by one examiner who had been calibrated following the manufacturer’s instructions for the use of the DIAGNOdent Pen;^20^ their inter-and intra-examiner agreement for LIF corresponded to a Cohen’s kappa coefficient of 0.89. The remineralizing agents were applied immediately after the LIF readings, firstly after the base-level measurement of the MIH defects and then every month for 12 months. Before performing the evaluation with the DIAGNOdent and during follow-up, the location of the lesion on the surface of the selected tooth was recorded on the data sheet; the possible locations were buccal, occlusal, occluso-buccal, occluso-palatal, occluso-distal, occluso-mesial, or bucco-mesial/distal surface.

### Statistical Analysis

All statistical analysis was carried out using the Stata 18.0 program (Stata; College Station, TX, USA). Frequencies, percentages, means and standard deviations were used to summarize the characteristics of the population. The Shapiro-Wilk test was used to assess whether the selected sample data set comes from a normally distributed population (LIF values). The paired-samples t-test was applied to data showing the differences between the initial measurement and the mean values obtained during the monitoring period. A repeated-measures ANOVA was used to compare the means of four groups when the same children were measured multiple times at different time points. The post-hoc Bonferroni test was used for the within-group comparison. The Greenhouse-Geisser (GG) correction was used in repeated measures ANOVA to adjust for violations of the sphericity assumption (equal variances). The GG correction adjusts the degrees of freedom to account for this violation and helps to prevent inflated p-values and a higher risk of Type I errors. All hypotheses were tested with significance set at p <0.05.

## RESULTS

The data that support the findings of this study are available from the corresponding author upon reasonable request.

### Description of the Study Population 

**Fig 2:**
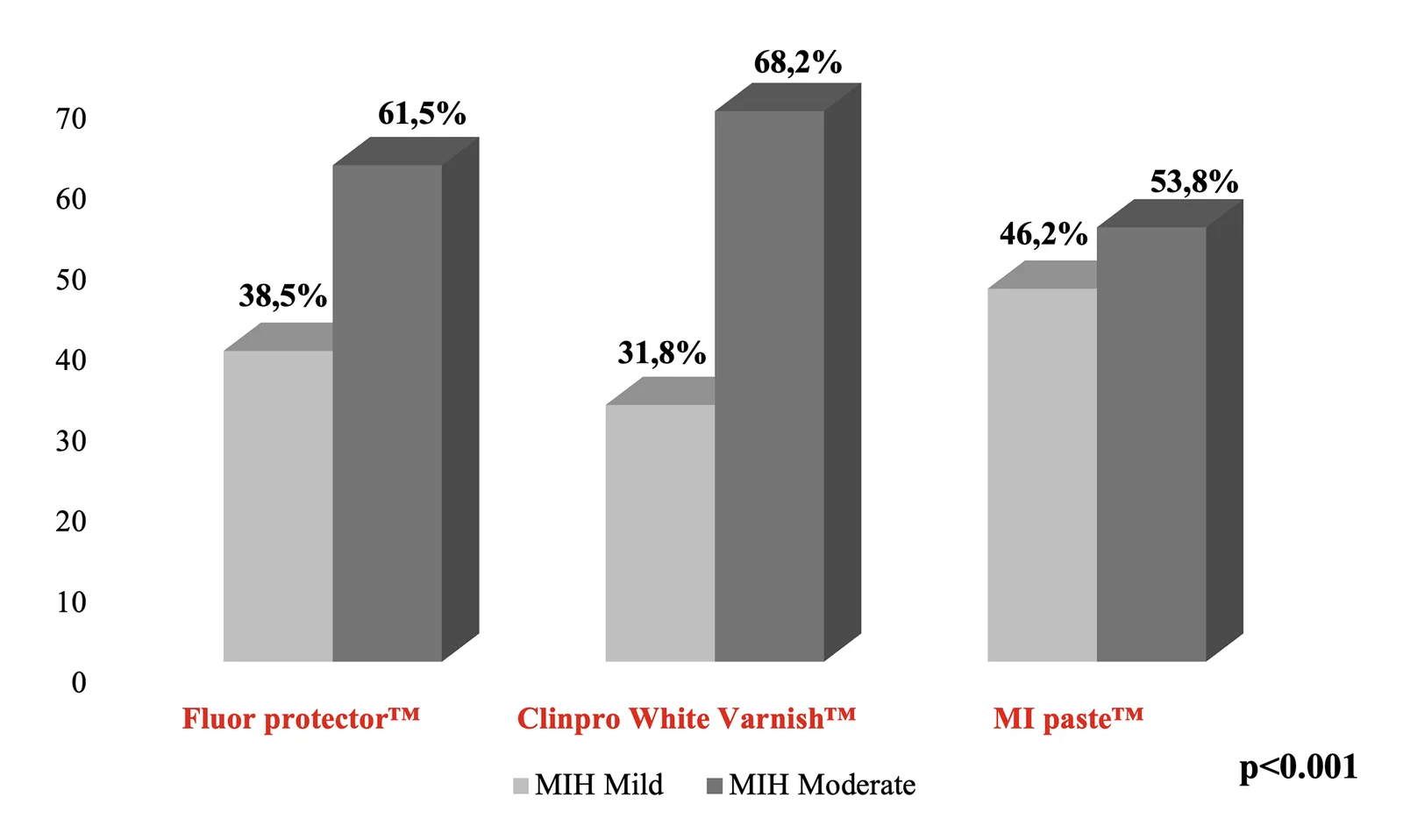
Percentage distribution of teeth with Molar Incisor Hypomineralization (MIH) by treatment group in Mexican schoolchildren.

A total of 104 children participated in the present study, of whom 58.0% were girls. The mean age of all participants was 9.50 ± 1.35 years. The sample comprised 78 children and a total of 168 teeth diagnosed with mild and moderate MIH (demarcated by creamy-white and yellow-brown lesions). Figure 2 presents the percentage distribution of the teeth with MIH by treatment group, with a similar distribution observed for the three groups.

Table 1 presents the measurements undertaken for the three treatment groups over a 12-month period, showing that the average LIF scores obtained at the beginning of the study were higher than those obtained at the 1-, 3-, 6-, 9-, and 12-month follow-up, for those teeth with both mild and moderate MIH, across all three treatment groups. While the remineralization effect was found to be minimal when comparing the basal evaluation with the values obtained after one month of treatment, from the third month onward, a statistically significant improvement was observed in the remineralization of teeth with both mild and moderate MIH across all three treatment groups (p<0.05).

**Table 1:** Comparison of means of laser fluorescence by severity of MIH and treatment group in Mexican schoolchildren aged 8-12 years, 12-month follow-up

Groups	Mild MIH														
	Baseline vs 1 month		Mean difference	Baseline vs 3 months		Mean difference	Baseline vs 6 months		Mean difference	Baseline vs 9 months		Mean difference	Baseline vs 12 months		Mean difference
	Mean (SE)			Mean (SE)			Mean (SE)			Mean (SE)			Mean (SE)		
Groups	Moderate MIH														
	Baseline vs 1st month		Mean difference	Baseline vs 3 months		Mean difference	Baseline vs 6 month		Mean difference	Baseline vs 9 months		Mean difference	Baseline vs 12 months		Mean difference
	Mean(SE)			Mean(SE)			Mean(SE)			Mean(SE)			Mean(SE)		
Fluor Protector	28.9(10.7)	23.2(8.1)	5.68**	28.9(10.7)	10.8(2.3)	18.0**	28.9(10.7)	9.2(2.8)	19.7*	28.9(10.7)	11.9(5.9)	16.9*	28.9(10.7)	9.8(4.2)	19.1*
Clinpro White Varnish	28.4(13.1)	21.8(10.7)	6.51**	28.4(13.1)	13.3(4.3)	15.0**	28.4(13.1)	12.3(4.1)	16.0**	28.4(13.1)	12.1(3.9)	16.2**	28.4(13.1)	9.0(3.3)	19.4**
MI Paste	35.4(13.0)	29.0(13.2)	6.39**	35.4(13.0)	21.1(9.5)	14.3**	35.4(13.0)	16.3(6.9)	19.2**	35.4(13.0)	13.7(3.1)	21.8**	35.4(13.0)	11.1(2.7)	24.3**
Fluor Protector	33.5(11.8)	26.1(10.3)	7.4**	33.5(11.8)	15.4(9.9)	18.1**	33.5(11.8)	16.1(7.9)	17.4**	33.5(11.8)	13.2(4.6)	20.2**	33.5(11.8)	10.2(3.7)	23.3**
Clinpro White Varnish	31.1(11.9)	23.1(10.6)	8.0**	31.1(11.9)	13.3(6.1)	17.8**	31.1(11.9)	13.8(3.8)	17.3**	31.1(11.9)	13.8(11.9)	17.3**	31.1(11.9)	10.3(2.5)	20.8**
MI Paste	42.5(8.5)	28.6(9.8)	13.9**	42.5(8.5)	19.9(7.8)	22.6**	42.5(8.5)	16.1(4.1)	26.4**	42.5(8.5)	13.1(2.0)	29.3**	42.5(8.5)	11.1(1.9)	31.4**
SE: standard error; paired samples t-test:*p<0.05, **p<0.001.															

The ANOVA applied to compare the repeated intergroup measures for the LIF values over the 12-month follow-up revealed changes in the averages over time for the three treatment groups, in contrast with the results obtained for the control group (p<0.001). The findings showed that the statistically significant differences in the LIF scores were due to the long-term effects of the remineralizing agents: F(6, 594) =117.39, p<0.001 (Table 2). In the general LIF values obtained, the post-hoc comparisons conducted on the changes observed over time show that the LIF of the three treatment groups decreased statistically significantly after the third month of follow-up. After the ninth month, a similar average decrease in the baseline LIF values was observed for the three treatment groups (p<0.05).

**Table 2:** Intergroup comparison of laser-induced fluorescence values in teeth with MIH over the study period in Mexican schoolchildren aged 8-12 years: a 12-month follow-up

Group	Baseline Mean (SD)	1 month Mean (SD)	3 months Mean (SD)	6 months Mean (SD)	9 months Mean (SD)	12 months Mean (SD)	p
Fluor Protector	31.3 (11.8)	24.6 (9.8)	13.8 (8.6)	13.9 (7.4)	12.6 (5.0)	10.0 (3.7)	<0.001
Clinpro White Varnish	29.9 (12.2)	22.6 (10.5)	13.3 (5.3)	13.2 (3.9)	13.1 (3.7)	9.8 (2.9)	
MI Paste	38.7 (11.5)	28.8 (11.6)	20.5 (8.7)	16.2 (5.7)	13.5 (2.6)	11.1 (2.3)	
Control	6.7 (1.5)	7.3 (0.8)	5.8 (0.8)	7.3 (0.8)	5.9 (0.8)	7.3 (0.8)	
SD: standard deviation.							

### Adverse Effects in the Study Population 

Over the course of the 12-month follow-up, the present study found neither adverse effects nor damage to the general and oral health of the children across all three treatment groups.

## DISCUSSION

The present clinical trial comprise a prospective longitudinal assessment of the effects of three remineralizing agents—Fluor Protector, Clinpro White Varnish, and MI Paste—on incisors and molars presenting mild and moderate MIH in Mexican children. After twelve months of follow-up, statistically significant decreases were found in the LIF scores obtained for all three treatment groups, decreases that were attributable to the remineralizing effects of the agents applied.

It has been widely acknowledged that the use of fluorides plays a fundamental role in the reduction and prevention of caries.^13^ Fluoride is a remineralizing agent that interacts with the oral fluids on tooth surfaces and combines with calcium and phosphate ions to form hydroxyapatite and fluorapatite, thus remineralizing the different areas of the enamel.^24^ In-vitro and in-vivo studies conducted on the remineralization of enamel/dentin have shown that high concentrations of fluorides reverse calcium loss in the enamel and dentin, thus enabling remineralization and inhibiting demineralization.^5^

The present study found that the use of Fluor Protector varnish had a remineralization effect on teeth with mild to moderate MIH during the 12-month follow-up. Fluoride varnishes are a valuable tool for remineralizing teeth and preventing caries, given that they release the fluoride ions that help to protect and remineralize tooth surfaces. Furthermore, the fluoride released by the varnishes increases fluoride concentration in the saliva, interacts with the hydroxyapatites of the enamel, interferes with bacterial metabolism, and accumulates on the tooth surface in the form of calcium fluoride.^25^ In-vitro studies have reported that fluoride varnishes increase the mineral density of the enamel,^4^ and Kim et al, using four varnishes (Duraphat, EnamelPro, MI, and Clinpro White), reported that fluoride varnishes present a remineralizing effect.^18^ Therefore, the application of fluoride varnish is a method with multiple advantages and is considered a viable, safte treatment option with minimal adverse effects for teeth damaged by MIH.

The mineral loss and reduced enamel hardness and elasticity in teeth with MIH can deteriorate the enamel and cause changes in tooth shape, function, and esthetic qualities, increasing sensitivity and leading to tooth loss.^32^ Fluoride varnishes have been used successfully to intervene in the demineralization/remineralization process, given their ability to increase enamel mineral density and eliminate tooth hypersensitivity.^31^ As described above, the remineralizing agents used in the present study—Fluor Protector, Clinpro White Varnish, and MI Paste—all presented a similar level of reduction in the average LIF values observed for mild and moderate MIH defects during the 12-month follow-up. While the literature contains few studies on the effect of remineralizing agents on teeth with MIH, Olgen et al^23^ evaluated the long-term efficacy of fluoride varnish and toothpastes that contain CPP-ACP in the remineralization of creamy-white and yellow-brown defects in permanent first molars with MIH. Those authors found that all remineralization agents increased remineralization rates in both creamy-white and yellow-brown colored defects without presenting any statistically significant difference at the end of the follow-up period. Although fluoride varnishes act more slowly than toothpastes containing CPP-ACP, they did increase the level of remineralization of MIH defects.^23^ Furthermore, Biondi et al^3^ evaluated mineral density posterior to the application of Duraphat, Clinpro, and CPP-ACP on mild and moderate MIH defects, finding that Clinpro was more effective on mild lesions, while Duraphat was found to be more effective on moderate MIH lesions. The present study found that the three remineralizing agents applied demonstrated similar efficacy in mild and moderate MIH lesions.

Finally, Kumar et al,^19^ in an in‑situ and in‑vitro study that applied a fluoride varnish and CPP-ACP on teeth with MIH, observed a statistically significant increase in calcium and phosphorous content, as well as a reduction in carbon content, over a period of six months, a finding which suggests a remineralization process occurring in both groups. Therefore, both fluoride varnishes (Fluor Protector and Clinpro White Varnish) and CPP-ACP are equally effective in achieving the remineralization of teeth damaged by MIH.

The present study used LIF to evaluate MIH lesions, observing a decrease in average LIF values over the course of twelve months for all three treatment groups. Recently, LIF has been used to evaluate MIH in clinical studies, presenting satisfactory clinics results.^3,23^ Enamel lesions produced by MIH can be evaluated both clinically^17^ and via the use of other methods, such as the DIAGNOdent Pen, which uses the LIF diagnostic method. The light absorbed by teeth is reflected in fluorescent form, with the intensity of the fluorescent light increasing in line with the depth of the lesion. Therefore, in teeth with MIH, the irregular structure of the enamel increases the quantity of fluorescence reflected by the tooth surface and, as a consequence, organic content increases and the mineral hardness of the enamel decreases.^3^ Moreover, studies have been conducted on the association between the mechanical properties of hypomineralized enamel and DIAGNOdent evaluations of teeth extracted due to MIH, finding that the increase in LIF values may be related to the proteins present in such hypomineralized enamel.^12^ Observed reductions in LF indicate successful remineralization and structural reinforcement of porous enamel. Clinically, these drops denote restored enamel hardness, reduced dentinal hypersensitivity, and a decreased risk of post-eruptive breakdown (PEB) in MIH teeth.

Currently, it has been reported that MIH likely occurs due to alterations in the calcification or maturation phase and has consequences on mechanical, physical, and morphological properties, such as increased porosity (cracks and deep pores), low hardness and elasticity, decreased mineral content, and reduced microhardness.^9^ In an in-vitro study using polarized Raman spectroscopy and scanning electron microscopy on teeth with MIH, Cardoso-Martins et al^7^ found greater mineral density and that the hypomineralized enamel was more organized after treatment with CPP-ACP.

Recent scientific findings on MIH have directly impacted preventive pediatric dentistry protocols and global-management as well as clinical guidelines in 3 major ways: 1) a shift from caries-risk to structural-risk stratification is taking place (asymmetric risk assessment, post-eruptive breakdown [PEB] predictions, evidence identifying hypomineralized second primary molars [HSPMs] as a strong predictor for MIH); 2) evolution of non-invasive remineralization and desensitization, instead of relying solely on traditional topical fluorides, updated protocols endorse combinations of CPP-ACP, tricalcium phosphate, and hydroxyapatite toothpastes; 3) modification of sealant and adhesion strategies (e.g., glass ionomer and resin infiltration).^30^

### Limitations

LIF evaluation undertaken with the DIAGNOdent Pen must be calibrated due to the inconsistent readings caused by the probes being worn down after a prolonged period of use, which occurs irrespective of the use of the personalized guide provided for the application of the device. As the use of toothpaste containing CPP-ACP is an intervention to be applied at home, results may have been influenced by the children’s ability to comply with the required application of the material throughout the 12-month follow-up. Without verifiable adherence data, researchers cannot distinguish between a treatment that is inherently ineffective and a treatment that simply was not performed correctly. On the other hand, blinding of the principal investigator was not possible due to differences in remineralizing agents. Incomplete blinding frequently introduces performance and detection bias. Unblinded assessors may subconsciously inflate or deflate reported treatment effects. In addition, the economic aspect of using CPP-ACP toothpastes must be considered: they are substantially more expensive than standard over-the-counter or prescription fluoride toothpastes. Finally, results from a single rural Mexican region may not be generalizable to other countries or high-caries regions. Furthermore, it is also necessary to control the fluoride intake from other sources.

## CONCLUSIONS

The use of three remineralizing agents—Fluor Protector, Clinpro White Varnish, and MI Paste—in children with mild and moderate MIH defects showed an increased long-term remineralization in evaluations conducted via laser-induced fluorescence. All remineralization agents increased remineralization rates in both creamy-white and yellow-brown colored defects without presenting any statistically significant differences betweem them at the end of the follow-up period. Fluoride varnish has the advantage that it is applied under clinical supervision in the dental office, while toothpaste containing CPP-ACP has the advantage of being used at home and therefore may reduce the need for dental visits. While any of the three remineralizing agents can be selected as a treatment option, the fact that fluoride varnish is less costly than toothpaste containing CPP-ACP should be taken into account. Finally, the results obtained can be used for the purposes of oral health prevention and improvement in the child and adolescent population, to help devise better treatment strategies that involve minimal intervention and will have a positive impact on quality of life.

## ACKNOWLEDGMENTS

We especially thank the Secretaría de Ciencia, Humanidades, Tecnología e Innovación (SECIHTI), for the support provided to carry out this project, Frontier Science (FS-2023-I-13). Thanks are also given to the Public Health Research Laboratory at the Iztacala Faculty of Higher Studies of the National Autonomous University of Mexico—particularly with the conceptual design of the study. The authors are very grateful to all the school-age children who participated in our study. The authors received financial support for the research, authorship, and/or publication of this article of Secretaría de Ciencia, Humanidades, Tecnología e Innovación (SECIHTI), (Frontier Science—FS-2023-I-13).
